# Lectures on the Theory and Practice of Physic

**Published:** 1841-01-30

**Authors:** 


### BIBLIOGRAPHICAL NOTICE.

*Lectures on the Theory and Practice of Physic.* By William Stokes, M. D., Lecturer at the Medical School, Park street, Dublin; Physician to the Meath Hospital, etc. etc. Second American edition. With numerous Notes, and twelve additional Lectures, by John Bell, M. D., Lecturer on the Institutes of Medicine and Medical Jurisprudence; Fellow of the College of Physicians of Philadelphia, etc. etc. Philadelphia: Haswell, Barrington & Haswell. 1840.

These lectures of Dr. Stokes are written in a clear, lively style, free from the dryness of most works on the practice of medicine, and yet so full of interesting matter that they received from the time of their publication in the Medical Gazette, an unusual share of attention. The popularity of these lectures was such that most of them were reprinted in the American Journal soon after their publication, and a complete series of them was afterwards republished in the American Medical Library. As this edition was a small one, the publishers of the present one soon felt themselves justified in putting another to press. This is rendered more valuable by a series of twelve lectures by Dr. Bell, the American editor, and by sundry notes dispersed through the text.

The style of Dr. Stokes's lectures is very familiar, and therefore extremely interesting; it is the true lecture style, which is not so well fitted for a systematic publication as for a course of lectures; neither is the work thrown into that methodical arrangement which renders it most convenient for reference: it is a book to read,—one that will be perused with great profit by the practitioner familiar with disease, and the student who has acquired a knowledge of the classification and relations of disease from more methodical writers; but it will not serve as a text book, to the exclusion of more regular treatises. It is, perhaps, this very peculiarity, which renders the work so popular: practitioners like as a general rule, to read works which come directly to the point, and dispense with the necessity of wading through much matter of inferior interest.

The additions of Dr. Bell comprise, besides his notes, twelve distinct lectures, on typhus, congestive, and eruptive fevers—rheumatism, acute and chronic--and chronic laryngitis. The subject most fully treated of is congestive fever, of which we may speak in terms of very high praise; it is very full, and will give a mass of useful information to the practitioner of the Southern States, where these diseases are so rife. Dr. Bell's visit to Italy, and familiarity with Italian medicine, have given him great advantages for this task, as no medical literature is more rich than that of the Italians in reference to this subject. A great part of Italy is proverbial for its unhealthiness and the prevalence of marsh fevers, and of course the attention of physicians has been as naturally directed to this matter, as that of the Parisian faculty to typhoid fever. The other additional lectures contain much valuable information, and enhance the merit of the work, with a single exception—that on typhus fever. We regret that this should have been inserted; it adds nothing of importance to the description of the disease given by Dr. Stokes; but, on the contrary, renders the subject more confused. It is very evident that the author never could have seen the two diseases to which he alludes in close connection, without avoiding the mistakes into which he has fallen. We quote the first paragraphs of his lecture:

"Dr. Stokes, in his remarks on the anatomical characters of fever, and the analogy of typhus with small-pox, does not attempt to separate, under two heads, typhus and typhoid fevers, as has been done by some zealous pathologists of late years. These latter tell us that typhus fever is an exanthematous disease, is contagious, and does not leave behind it any uniform anatomical lesion or altered structure of organs: typhoid fever, on the other hand, is not contagious, does not uniformly or characteristically exhibit an eruption, but has, as a constant character, an anatomical lesion, which consists in an alteration, by inflammation and ulceration of the glands of Peyer and Brunner, and inflamed mesenteric glands. The initial symptoms of the two diseases differ in the earlier and greater stupor and suffusion of the eyes in typhus. Some of the French pathologists have proposed to designate typhoid fever by its anatomical character, and it is by some called *dothinenteritis*, or *follicular enteritis*. But a little inquiry will make us backward in assenting to the accuracy of this division, and of the distinctions on which it rests. Thus, it is known that typhoid fever has commonly an eruption, which is stated expressly by M. Chomel to be one of its peculiarities, and to appear between the seventh and ninth days of the disease;—and it is accordingly mentioned in my long note on this subject, page 101. *Petechiæ* and *sudamina* are clearly understood by M. Andral to be diagnostic symptoms. It is true that the petechiæ of typhoid are chiefly confined to the abdomen and anterior parts of the chest, and they are of a rose colour: those of typhus are for the most part general, and are of a slightly livid or purple tint.

"But, as regards the anatomical character of typhoid fever, we learn from the same excellent and impartial authority, M. Andral, that patients have perished under this fever with all its symptoms well marked; yet still there was neither exanthema, certainly no ulcerations nor appreciable alteration in any part of the digestive tube. Can the proposition be inverted, and shall we be told that as there is typhoid fever without dothinenteritis, so there may be dothinenteritis without typhoid fever? This is the fact, since follicular enteritis has been found in other diseases, such as phthisis, scarlatina, remittent fevers, diarrhœa, and cholera. It were superfluous after this, to say that this variety of enteritis may go through its stages without giving rise to the phenomena of the fever called typhoid. Indeed, M. Louis himself, the strongest advocate for the anatomical lesion of the intestines as constituting the fixed character of the fever, tells us that headach, which occurs in forty-nine out of fifty cases of fever, is not met with in two out of the same number of enteritis. The rose-lenticular spots, the sudamina, epistaxis, tympanites, so common in typhoid fever, are rare in enteritis. This last disease may occur in infancy, may be repeated several times, and may complicate other diseases; whereas typhoid fever rarely attacks very young or very old individuals."

It is very possible that "some zealous pathologists," as Dr. Bell is pleased to style them, may not have been clear enough in their descriptions of the disease; but we cannot understand how one disease, which does not present a lesion which is either invariably or so often met in another that the exceptions are all doubtful, should be identified with it. Nor do we think it just that the error committed by so excellent a pathologist as Dr. Andral should be quoted, almost in reproach to him. His statement is simply, that a disease presenting all the characters of typhoid fever, was found without either exanthema or disease of the intestines; just as if a disease could present all the characters of pneumonia without either cough or inflammation of the lung. Of course this is a contradiction in terms; the cough may be latent, but the inflammation of the lungs is there, and the anatomical character is fixed. Dr. Andral confounded typhoid fever with what he calls the typhoid state,—that is, a group of symptoms, to which the terms adynamic, malignant, &c., are given, which occur in all cases in which there is a peculiar alteration of the blood, with inflammatory action, as phlebitis, metastatic abscess, &c. But the two diseases, typhoid and typhus fever, co-exist at the same time; one may be epidemic, as at Philadelphia, and the other sporadic,—yet in no instance of several hundred cases of epidemic typhus, and of some forty or fifty of sporadic treated at the same time in the Philadelphia Hospital, did we observe one disease converted into the other. This was true both of the mild and severe cases.

The notes attached to different lectures are generally very appropriate, and add materially to the character of the work, making it much more perfect; some of these notes are considerable enough to claim a place with the "additional lectures."

